# Supplementary material for: Chromothripsis during telomere crisis is independent of NHEJ, and consistent with a replicative origin
Source: Genome Res. 2019 May;29(5):737–49. doi: 10.1101/gr.240705.118 (PMC6499312; doi:10.1101/gr.240705.118)
Supplement: Supplemental Material [file supp_gr.240705.118_Supplemental_file_1.zip › contigs/annotated_contigs/DB37/contig.2.DB37_length_444_mean_cov_7.34234234234.docx]

**DB37_length_444_mean_cov_7.34234234234**

```
GAAATAAAGACAGATCATGTACAAAGAATAGTCATTAGACTGAAGACCTACTCCTCAACAGCAACAATAGACACCTGAAGCCAAAACCT
 >chr9:73463498-73463612 + E=5e-57 p=0e+00
TCCATGTGTC|AAAAGAAATTACCTG|TCAGTCTAGAATTCTACATTCAGCAAAACTATCTTTTAAAAATGAGAGCAGATTATAGATAT
           >chr9:73463913-73464255 + E=6e-168
TTTCATACTGCCAAAACTGACAGAGTTTACTTCTAGGCACCTTCACTGAAGGAAGTTGTTTTTGTTATTGTTTTTGTTTGTTTGTTTTG

AGATAGAGTCTTGCACTGTTGCCTGGGCTGGAACACAATGGCATGATCTCGGCTTACTGCAACCTTTGCCTCCTGGGTTCAAGCGATTC

TCCTGCCTCAGCCTCCCAAGTAGCTGGGACTACAGGCGCCCGCCACCACGCCCGGCTAATTTTTTTTTTGTATTTTTAGTAGAGACGGG

G
```
